# Determination of glucose cut-off points for optimal performance of glucagon stimulation test

**DOI:** 10.3389/fendo.2024.1448467

**Published:** 2024-08-28

**Authors:** Joanna Kawalec, Wojciech Horzelski, Małgorzata Karbownik-Lewińska, Andrzej Lewiński, Krzysztof C. Lewandowski

**Affiliations:** ^1^ Department of Endocrinology & Metabolic Diseases, Polish Mother’s Memorial Hospital Research Institute, Lodz, Poland; ^2^ Faculty of Mathematics and Computer Science, University of Lodz, Lodz, Poland; ^3^ Department of Endocrinology & Metabolic Diseases, The Medical University of Lodz, Lodz, Poland; ^4^ Faculty of Medicine, Mazovian University in Plock, Plock, Poland

**Keywords:** glucagon stimulation test, growth hormone, cortisol, glucose, cut-off point, pituitary, pituitary function

## Abstract

**Introduction:**

The glucagon stimulation test (GST) is widely used to assess growth hormone (GH) and cortisol secretion, nevertheless the precise mechanisms underpinning these hormonal responses remain unclear. We have endeavoured to explore the relationship between glucose and insulin fluctuations during GST and their impact on GH and cortisol secretion.

**Subjects and methods:**

We retrospectively studied 139 subjects (mean age 35.5 ± 15.1 years, BMI 26.6 ± 6.61 kg/m²), including 62 individuals with a history of pituitary disease (27 with an intact adrenal axis) and 77 healthy controls. Standard dose intramuscular GST was performed in all subjects.

**Results:**

Once BMI and age were excluded from multivariate model, the nadir of glucose concentration during GST was the sole variable associated with maximal GH secretion (ΔGH, p<0.0003), while neither glucose/insulin peak, nor Δglucose/Δinsulin concentrations contributed to ΔGH. 100% pass rate for GH secretion above 3 ng/ml or 1.07 ng/ml cut-offs was observed for glucose concentrations at, or below 60 mg/dl (3.33 mmol/l) (for Controls), or 62 mg/dl (3.44 mmol/l) (for Controls and patients with an intact adrenocortical axis). Such low glucose concentrations were obtained, however, only in about 30% of studied individuals. Conversely, cortisol secretion did not correlate with glucose or insulin fluctuations, suggesting alternative regulatory mechanisms.

**Conclusions:**

This study reveals that glucose nadir below 3.33 mmol/l is the only biochemical biovariable linked with optimal GH secretion during GST, whereas mechanisms responsible for cortisol secretion remain unclear. We emphasize the importance of glucose monitoring during GST to validate GH stimulation and support clinical decisions in GH deficiency management.

## 1 Introduction

Though an insulin tolerance test (ITT) is considered the gold standard for assessment of the integrity of the hypothalamic-pituitary-adrenal (HPA) axis, the test requires intensive medical and nursing supervision (1). It is also recognized that ITT is unpleasant for patients and contraindicated in those with ischaemic heart disease and epilepsy, as well as not advised in children and in the elderly (1). Furthermore, despite the status of the “gold standard”, ITT cortisol concentration cut-offs still appear to be the subject of debate (2). Hence, glucagon stimulation test (GST) is one the most popular alternative tests employed for many years in the assessment of HPA axis, as well as in the assessment of growth hormone (GH) secretion (3). Interestingly, these effects are observed after intramuscular or subcutaneous but not after the intravenous route of glucagon administration (4). Recently an intranasal glucagon administration was also described (5), where there was some stimulation of cortisol and GH secretion, though not strong enough to be applicable for clinical practice. Apart from transient nausea the test appears to be well tolerated (6), though occasional hypotension was reported in elderly people (7). Insulin-induced hypoglycaemia, utilized for many years in assessment of cortisol and GH secretion during ITT, has been considered as a driving factor behind GH and cortisol secretion (8, 9). Glycaemic fluctuations have also been implicated in stimulation of GH secretion during GST (10), though frank hypoglycaemia (i.e. below 40 mg/dl (2.2 mmol/l)) is not observed after intramuscular glucagon. The data derived from ITT suggest, however, that such severe hypoglycaemia is not always necessary to obtain adequate cortisol and GH responses as clinically symptomatic hypoglycaemia is as effective as biochemically confirmed hypoglycaemia during the ITT (11).

On the strength of these data we have endeavoured to formally assess a relationship between glucose and insulin fluctuations during GST and dynamics of cortisol and GH secretion.

## 2 Subjects and methods

This retrospective study included 139 subjects (43 males), age 35.5 ± 15.1 (mean ± SD), range 8–84, BMI 26.6 ± 6.61 kg/m^2^, range 14.5–44,7, divided into subjects with history of pituitary disease (n=62 – PITUITARY PATIENTS, 34 females) and controls (n=77 – CONTROLS, 63 females). Control group was recruited from medical staff of our Department as well as from patients with an intact pituitary function, but admitted with pituitary-unrelated conditions, e.g. irregular periods (differential diagnosis of PCOS, pituitary-related causes excluded), non-toxic goitre or tiredness (usually referred as “suspected endocrine disease”). Patients with history of pituitary disease were on average older (mean 42.3 years versus 29.8 years, p<0.001, and had slightly higher BMI (mean 29.1 kg/m^2^ versus 24.5 kg/m^2^, p=0.0006, t-test). Notably, in the Control group there were only four subjects aged less than 18 and a single subject aged eight. Three individuals (two Patients and one Control) had BMI above 40 kg/m^2^, while there were three individuals (all Controls) with BMI below 17.5 kg/m^2^.

Etiologies of patients with pituitary disease were as follows - pituitary adenoma: n=50, isolated diabetes insipidus: n=4, one case of hypopituitarism with craniopharyngioma (on desmopressin), genetic disorders causing pituitary dysfunction (PROP1 and POU1F1 mutations: n=3, two cases of congenital hypopituitarism without specific mutation investigated for), Rathke’s cleft cyst: n=2. Known gonadal axis deficiency was present in 21 subjects (no estrogen hormone replacement was taken in females prior to testing, while seven males receive intramuscular testosterone and one received hCG and recombinant FSH injections, while seeking fertility). Known thyroid axis deficiency was present in seven subjects (on L-thyroxine replacement).

In all subjects glucagon (GlucaGen 1 mg HypoKit^®^, Novo Nordisk, Denmark) was administered intramuscularly after an overnight fast at the dose of 1 mg or 1.5 mg depending on patients body mass, i.e. 1.5 mg for those over 90 kg. Cortisol, glucose, insulin and GH concentrations were assessed according to GST “short protocol”, i.e. at 0–60-90–120-150–180 minutes. Exclusion criteria to GST included diabetes mellitus and hyponatremia (plasma sodium below 136 mmol/l).

Serum GH concentrations were measured using the immunochemiluminescence assay IMMULITE 2000 Xpi^®^ (Siemens, Munich, Germany), interassay variation 3%, intraassay variation 2.3%. Serum cortisol and insulin were measured by the means of electrochemiluminescence assay Elecsys Cortisol II on Cobas 6000 platform (Roche, Basil, Switzerland), interassay variation 3.3%, intraassay variation 2.6%.

Integrity of the HPA axis was confirmed, if any cortisol concentration during GST was above 13.6 µg/dl (>375 nmol/l) as suggested by Yo WS, et al. (12). The cutoff value for a successful GST was defined as GH>3 ng/ml (13) though we also assessed the data when using a lower GH cut-off during GST, i.e. 1.07 ng/mL, as suggested by Diri H, et al. (14).

### 2.1 Statistical analysis

The MedCalc 19.0.7 package was used for statistical analysis. Shapiro-Wilk and D’Agostino-Pearson tests were used to test the normality of distributions. The t-Student and Mann-Whitney methods were used to compare parameters (after applying the Fisher-Snedecor test). Variance analysis (ANOVA) was used to compare more parameters. Spearman’s rank correlation coefficient was used to determine the correlation between parameters. The analysis for serial measurements was also applied (Wilcoxon signed-rank test). The P<0.05 ratio was taken as statistically significant.

The study has been approved by the Ethics Committee of The Polish Mother’s Memorial Hospital Research Institute, decision 63/2020. All patients provided written permission that their data might be presented anonymously for research and training purposes in accordance with Regulation (*EU*) 2016/679 of the European Parliament and of the Council of 27 April 2016 on the protection of natural persons with regard to the processing personal data and on the free movement of such data.

## 3 Results

**Figure 1:**
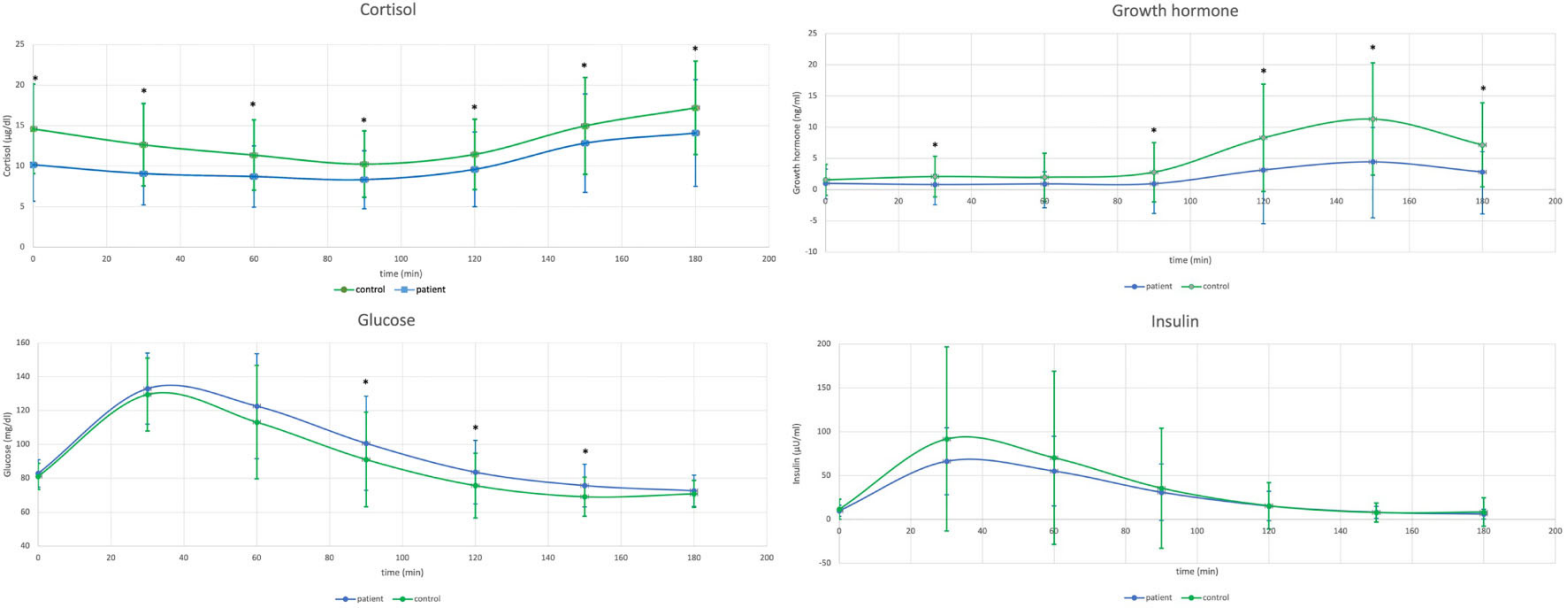
Comparison of cortisol concentrations during glucagon stimulation test (GST) in healthy controls (n=77) versus patients with a history of pituitary disease (n=62) - upper left, and comparison of growth hormone concentrations during GST in healthy controls versus patients with a history of pituitary disease - upper right. Lower left: Comparison of glucose concentrations during GST in healthy controls (n=77) versus patients with a history of pituitary disease (n=62), and lower right: comparison of insulin concentrations during GST in healthy controls versus patients with a history of pituitary disease.

Comparison of GH, cortisol, glucose and insulin within the Pituitary Patients and Controls at all time points are presented in **Table 1**, **Figure 1**.

**Table 1:** Comparison of cortisol, growth hormone, glucose and insulin concentrations between patients with a history of pituitary disease (n=62) and Controls with an intact pituitary function (n=77).

CORTISOL [µg/dl]	PATIENTS, n=62		CONTROLS, n=77		p
	MEAN	SD	MEAN	SD	
0 MIN	10.17	4.51	14.60	5.53	p < 0.001
30 MIN	9.08	3.86	12.64	5.09	p < 0.001
60 MIN	8.71	3.78	11.37	4.34	0.0004
90 MIN	8.33	3.58	10.26	4.09	0.0043
120 MIN	9.60	4.61	11.46	4.35	0.0164
150 MIN	12.84	6.06	14.97	5.96	0.0386
180 MIN	14.09	6.59	17.20	5.78	0.0036
GROWTH HORMONE [ng/ml]					
0 MIN	1.01	2.26	1.57	2.47	0.1753
30 MIN	0.81	1.63	2.10	3.23	0.0049
60 MIN	0.92	1.95	1.99	3.83	0.4999
90 MIN	0.95	1.72	2.79	4.76	0.0044
120 MIN	3.15	5.11	8.30	8.59	0.0001
150 MIN	4.45	5.49	11.31	8.97	p < 0.001
180 MIN	2.82	3.24	7.18	6.72	p < 0.001
GLUCOSE [mg/dl]					
0 MIN	82.90	8.12	81.18	7,76	0.2050
30 MIN	133.00	21.04	129.50	21.57	0.3394
60 MIN	122.69	31.03	113.23	33.54	0.0913
90 MIN	100.73	27.73	91.16	27.97	0.0475
120 MIN	83.68	18.77	75.76	19.16	0.0162
150 MIN	75.76	12.65	69.16	11.55	0.0019
180 MIN	72.72	9.21	70.83	7.94	0.1981
INSULIN [µU/ml]					
0 MIN	9.92	6.31	11.69	11.48	0.2824
30 MIN	66.41	38.12	91.77	104.98	0.0802
60 MIN	55.19	39.68	70.37	98.64	0.2645
90 MIN	31.03	32.21	35.66	68.37	0.6282
120 MIN	15.37	16.70	15.63	26.44	0.9479
150 MIN	8.18	6.88	7.94	10.74	0.8839
180 MIN	6.26	5.59	8.45	16.14	0.3170

Statistically significant differences are highlighted in red.

Patients with history of pituitary disease had lower cortisol concentrations at all time-points and lower GH concentrations with exception of times 0 and 60 minutes. On the contrary, glucose concentrations were higher in patients with history of pituitary disease at 90, 120 and 150 minutes of GST. At all time-points there were no significant differences in insulin concentrations between investigated groups (**Table 1**), and there were also no differences in glucose x insulin product (HOMA-IR equivalent, p ranging from 0.1 at 30 minutes to 0.76 at 90 minutes of GST, data not shown). Results of longitudinal comparisons within groups are presented in **Table 2**. There was an initial significant increase in glucose and insulin at 30 and 60 minutes of GST, followed by transient return to initial values, either at 90 or 120 minutes (for glucose), or at 120 minutes (for insulin), followed by subsequent fall in both glucose and insulin below initial concentrations observed at 0 minutes. This was accompanied by a significant rise in GH concentrations (at 120, 150 and 180 minutes of GST), with peak GH concentrations at 150 minutes of GST (difference 150 versus 180 minutes, p=0.0026, significant for Controls only). In case of cortisol, there was an initial fall in cortisol concentrations followed by significant rise at the end of GST (most pronounced at 180 minutes). All Controls had at least a single cortisol concentration above 13.6 ug/dl during GST, however, eight subjects (10.4%) failed to achieve 3 ng/ml GH cut-off, while four subjects (5.2%) failed to achieve 1.07 ng/ml GH cut-off. Fully intact adrenal axis was found in 27 subjects (43.5%) with a history of pituitary disease, while suboptimal GH secretion was observed for 31 (50%) and 20 (32.2%) for 3 ng/ml and 1.07 ng/ml cut-off, respectively.

**Table 2:** Longitudinal comparison of insulin, glucose, cortisol and growth hormone concentrations in Controls (n=77) and Patients with history of pituitary disease (n=62) within each group.

		0’ vs 30’	0’ vs 60’	0’ vs 90’	0’ vs 120’	0’ vs 150’	0’ vs 180’
**INSULIN [µU/ml]**	**PATIENT**	< 0.0001	< 0.0001	< 0.0001	0.2221	0.0346	<0.0001
	**CONTROL**	<0.0001	<0.0001	0.0006	0.0658	<0.0001	<0.0001
**GLUCOSE [mg/dl]**	**PATIENT**	< 0.0001	< 0.0001	0.0003	0.4502	0.0002	< 0.0001
	**CONTROL**	<0.0001	<0.0001	0.1703	0.0002	<0.0001	<0.0001
CORTISOL* **[µg/dl]**	**PATIENT**	0.1061	0.0369	0.0075	0.3373	0.0058	0.0002
	**CONTROL**	0.0427	<0.0001	<0.0001	0.0002	0.8298	0.0087
**GROWTH HORMONE** **[ng/ml]**	**PATIENT**	0.2398	0.5317	0.2633	0.0001	<0.0001	<0.0001
	**CONTROL**	0.7944	0.7944	0.1677	<0.0001	<0.0001	<0.0001

*Both in Patients and Controls cortisol concentrations at 30, 60, and 90 minutes were significantly lower than at 150 and 180 minutes, p<0.0001.

Statistically significant differences are highlighted in red, for precise numerical data see **Table 1**.

More detailed analysis of cortisol, GH, glucose and insulin fluctuations for all GST time-points combined is presented in **Table 3**. Though average cortisol concentrations (both minimal and maximal) were lower in patients with pituitary disease (as mentioned above at least 21 of these patients had various degrees of hypopituitarism) with greater cortisol decline versus 0 minutes in Controls (p<0.0002), overall fluctuations of cortisol concentrations (Δ i.e. maximal minus minimal value) was similar in both groups (p=0.1856). This contrasted with fluctuations in GH concentrations, where Controls had both higher maximal concentrations and greater GH fluctuations (Δ). In Control group we observed lower minimal glucose concentrations (63.46 ± 9.82 mg/dl versus 69.14 ± 10.46 mg/dl, p=0.0013, for Controls and Pituitary Patients, respectively) and greater decline against glucose concentrations at the beginning of GST (18.54 mg/dl ± 12.57 mg/dl versus 13.54 ± 8.23 mg/dl, p=0.0074, for Controls and Pituitary Patients, respectively). There were, however, no differences in both maximal glucose concentrations and in degree of glucose fluctuations (Δ glucose, p=0.98). In contrast, Controls had significantly higher maximal insulin concentrations (p=0.047) and significantly greater insulin fluctuations during GST (p=0.04).

**Table 3:** Comparative analysis of cortisol, growth hormone, glucose and insulin fluctuations between patients with a history of pituitary disease (n=62) and healthy controls (n=77) (Mann-Whitney U test).

CORTISOL [µg/dl]	PATIENT, n=62		CONTROL, n=77		p
	MEAN	SD	MEAN	SD	
Minimal value	7.04	3.09	8.89	4.03	0.0040
Maximal value	15.43	6.49	18.45	5.32	0.0036
DECLINE versus 0’ min	3.31	3.19	6.06	4.97	0.0002
Δ (maximal minus minimal value)	8.29	5.71	9.56	5.39	0.1856
GROWTH HORMONE [ng/ml]					
Minimal value	0.45	1.14	0.59	1.27	0.4927
Maximal value	5.10	5.77	13.76	9.27	<0.0001
DECLINE versus 0’ min	0.54	1.34	0.94	1.68	0.1216
Δ (maximal minus minimal value)	4.65	5.56	13.17	9.27	<0.0001
GLUCOSE [mg/dl]					
Minimal value	69.15	10.46	63.46	9.82	0.0013
Maximal value	137.65	24.10	130.66	27.75	0.1211
DECLINE versus 0’ min	13.54	8.23	18.55	12.57	0.0074
Δ (maximal minus minimal value)	68.50	23.37	68.43	24.29	0.9872
INSULIN [µU/ml]					
Minimal value	5.56	4.80	8.88	27.52	0.3544
Maximal value	69.86	40.27	98.92	106.98	0.0470
DECLINE versus 0’ min	4.22	3.77	2.86	27.53	0.6969
Δ (maximal minus minimal value)	64.30	37.85	93.12	102.62	0.0400

Statistically significant differences are highlighted in red.

Correlation analysis is presented in **Table 4A** and revealed that increase of cortisol concentrations during GST, i.e. Δ Cortisol, correlated only with a degree of GH increase (ΔGH), but there was no correlation with any of glucose and insulin variables. In contrast, Δ GH correlated with minimal glucose and insulin concentrations (r=-0.308, p=0.0003, r=-0.267, p=0.0018, for glucose and insulin, respectively) (**Table 4A**). There was, however, no significant correlation between Δ GH and both Δ glucose and Δ insulin (**Table 4B**). These observations were valid, even if individuals with extremes (BMI <17.5 kg/m^2^, or BMI>40 kg/m^2^, n=6) were eliminated from the analysis, or after elimination of subjects aged less than 18 (n=4).

**Table 4A:** Univariate Spearman rank correlation analysis of fluctuations of GH, cortisol, glucose and insulin (maximal minus minimal concentration, i.e. Δ) during glucagon stimulation test for the whole group (n=139).

	glucose min.	glucose max	insulin min	insulin max	HGH min	HGH max	Cortisol min	Cortisol max
**Δ Cortisol**	r=-0.103 p=0.24	-0.107 0.2198	-0.101 0.2569	0.133 0.1305	0.106 0.2252	0.266 0.0020	-0.150 0.0838	0.765 <0.0001
**Δ GH**	r= -0.308 p=0.0003	-0.180 0.3555	-0.267 0.0018	-0.024 0.7833	0.498 <0.0001	0.988 <0.0001	0.162 0.0626	0.312 0.0003
**Δ insulin**	r=-0.051 p=0.5552	0.050 0.5656	0.390 <0.0001	0.996 <0.0001	-0.142 0.1010	-0.017 0.8495	-0.139 0.1130	0.030 0.7421
**Δ glucose**	r= -0.187 p=0.0276	0.866 <0.0001	0.263 0.0021	0.076 0.3804	-0.215 0.0115	-0.064 0.4603	-0.002 0.9788	-0.053 0.5450

Statistically significant differences are highlighted in red.

**Table 4B:** Univariate Spearman rank correlation analysis of relationship between maximal minus minimal concentrations of cortisol, GH, glucose and insulin Δ during glucagon stimulation test for the whole group n=139.

	Δ GH	Δ insulin	Δ glucose
**Δ Cortisol**	r=0.2760 (p= 0.0013)	0.1510 (0.0842)	-0.0290 (0.7408)
**Δ GH**	–	-0.0042 (0.9613)	-0.0293 (0.7342)
**Δ insulin**	–	–	0.0599 (0.4920)

Statistically significant concentrations are highlighted in red.

In multivariate analysis, however, there was no significant variable that correlated with cortisol increase (Δ Cortisol). In relation to Δ GH, BMI and age, were the only variables related to GH increase (**Table 5A**). However, when age and BMI were eliminated from the model, minimal glucose concentrations represented the only variable that correlated with Δ GH (p=0.0003, **Table 5B**).

**Table 5A:** Multivariate analysis of variables related to GH increase (ΔGH) with inclusion of age and BMI in the model.

Independent variables	Coefficient	Std. Error	P	R
(Constant)	29.434			
AGE	-0.1117	0.0545	0.043	-0.1877
BMI_kg/m²_	-0.5793	0.1307	<0.001	-0.3819
glucose_min.	-0.0338	0.0837	0.4871	-0.0876
Δ_glucose	0.0179	0.0315	0.5706	0.0530
insulin_min	0.0478	0,1974	0.8088	0.0226
Δ_insulin	-0.0031	0.0116	0.7912	-0.0247

Statistically significant differences are highlighted in red.

**Table 5B:** Multivariate analysis of variables related to GH increase ΔGH after exclusion of BMI and age from the model, here only nadir of glucose concentrations remains significant:

Independent variables	Coefficient (R)	Std. Error	P
(Constant)	26,4276		
GLUCOSE_min.	-0.2581	0.07247	0.0005
Other Variables included into the model, but without statistical significance			
insulin_max			
insulin_min			
Δ insulin			
glucose_max			
Δ glucose			

Statistically significant variables are highlighted in red.

In another step, in the Control group, we analyzed what minimal glucose concentration should be achieved during GST, so that at least a single GH concentration during GST would be either above 3.0 ng/ml, or above 1.07 ng/ml (n=77 – **Table 6A**). We have repeated that analysis for patients with intact adrenal axis in order to eliminate subjects with significant GH deficiency, i.e. in a combined group of subjects with an intact adrenal axis (at least one cortisol concentration during GST above 13.6 µg/dl (>375 nmol/l), n=104 – **Table 6B**). None of these subjects took any hormonal replacements. Notably if 500 nmol/l (18 µg/dl) cortisol cut-off were applied then 35 out of 62 Patients (56%), but also 21 Control subjects (27%) failed to reach this threshold.

**Table 6A:** Analysis of glucose [mg/dl or mmol/l] threshold above which at least a single GH concentration would be either above 1.07 ng/ml (14) or above 3.0 ng/ml (13) thus denoting adequate GH secretion during GST in the Control group (n=77).

CONTROLS (n=77)		
	GH > 1.07 ng/ml	GH > 3 ng/ml
Sample size	73 out of 77 (94.8%)	69 out of 77 (88.6%)
Lowest value	44 mg/dl**/**2.44 mmol/l	44 mg/dl**/**2.44 mmol/l
Highest value	93 mg/dl**/**5.17 mmol/l	93 mg/dl**/**5.17 mmol/l
Median	63.5 mg/dl/3.53 mmol/l	63.5 mg/dl/3.53 mmol/l
95% CI for the median	61.2 to 65.8 mg/dl**/**3.4-3.65 mmol/l	60.4 to 65.6 mg/dl**/**3.36-3.64 mmol/l
Interquartile range	58.5 to 69 mg/dl**/**3.25-3.83 mmol/l	58.5 to 68.5 mg/dl**/**3.25-3.81 mmol/l
100% sensitivity glucose cut-off	≤60 mg/dl (3.33 mmol/l) (n=23, 29.9%)	≤60 mg/dl (3.33 mmol/l) (n=23, 29.9%)

This implies 100% sensitivity for adequate GH secretion as long as the trough of glucose concentrations is at, or below 60 mg/dl (3.33 mmol/l). Statistically significant differences are highlighted in red.

**Table 6B:** Analysis of glucose threshold mg/dl or mmol/l above which at least a single GH concentration would be either above 1.07 ng/ml 14 or above 3.0 ng/ml 13 thus denoting adequate GH secretion during GST.

Controls, n=77 + 27 of Patients with history of pituitary disease and intact adrenal axis, n=104		
	GH > 1.07 ng/ml	GH > 3 ng/ml
Sample size	92 out of 104 (88.5%)	85 out of 104 (81.7%)
Lowest value	44 mg/dl**/**2.44 mmol/l	44 mg/dl**/**2.44 mmol/l
Highest value	97 mg/dl**/**5.39 mmol/l	97 mg/dl**/**5.39 mmol/l
Median	65 mg/dl/3.61 mmol/l	64 mg/dl/3.56 mmol/l
95% CI for the median	63 to 66.9 mg/dl**/**3.5-3.72 mmol/l	63 to 66 mg/dl**/**3.5-3.67 mmol/l
Interquartile range	59 to 70 mg/dl**/**3.28-3.89 mmol/l	59 to 70 mg/dl**/**3.28-3.89 mmol/l
100% sensitivity glucose cut-off	≤62 mg/dl (3.44 mmol/l) N=33 (31.7%)	≤62 mg/ml (3.44 mmol/l) N=33 (31.7%)

Analyzed group included subjects with an intact adrenal axis, i.e. at least one cortisol concentration during GST above 13.6 µg/dl >375 nmol/l 12, who did not take any hormonal supplements n=104. This implies 100% sensitivity for adequate GH secretion as long as the trough of glucose concentrations is at, or below 62 mg/dl 3.44 mmol/l. Statistically significant differences are highlighted in red.

In Control subjects 100% sensitivity, both for peak GH concentrations above 1.07 and above 3 ng/ml during GST, was obtained for glucose concentration at or below 60 mg/dl (3.33 mmol/l). Such low glucose concentration was, however, obtained only by 23 subjects (29.9%).

The corresponding 100% sensitivity glucose concentration cut-offs for the combined group of subjects with an intact adrenal axis (n=104) were 62 mg/dl (3.44 mmol/l), both for GH cut off of 1.07 ng/ml and 3.0 ng/ml. Such low glucose concentration during GST, was, however, obtained only in 33 (31.7%) of investigated subjects. That implies that 10 out 27 patients with an intact HPA axis (37%) managed to obtain glucose concentrations equal or below 62 mg/dl. Four of those (15%) who failed to obtain glucose nadir at, or below 62 mg/dl failed to reach 3.0 ng/ml GH cut off, while all obtained GH secretion above 1.07 ng/ml cut-off.

## 4 Discussion

Our study demonstrates that, once BMI and age are excluded, then, among those analyzed, the nadir of glucose concentrations during GST is the sole independent variable associated with an increase in GH concentrations (ΔGH). In contrast, other parameters, such a degree of glucose (i.e. Δ glucose) or insulin fluctuations have no impact on either GH, or cortisol release during GST. In particular Δ glucose was not related to either peak GH secretion, or to Δ GH. This is consistent with results obtained by Wilson et al. (15), where glucose nadir was inversely related to GH area under the curve (rs=-0.38; p=0.03) and GH peak (rs=-0.37; p=0.04). Wilson et al. (15) did not investigate a relationship between glucose and insulin fluctuations and cortisol secretion. Furthermore, in contrast to their study, we simultaneously measured both cortisol and GH responses during GST in much larger number of subjects (139 versus 42), and provided calculated data on an optimal glucose concentration, below which we can expect satisfactory GH response to intramuscular glucagon in subjects with an intact anterior pituitary function, i.e. 60 mg/dl (3.33 mmol/l), for both 3.0 ng/ml and 1.07 ng/ml GH cut-offs.

This implies that glucose concentrations should be measured during GST in order to validate obtained GH concentrations. We note, however, that most healthy subjects (i.e. 64.9% and 59.7%, for 1.07 ng/ml and 3.0 ng/ml GH cut-off, respectively) manage to obtain satisfactory GH response despite higher glucose nadir. In a similar way many patients show satisfactory GH response during ITT, even if glucose concentration fails to fall below 40 mg/dl (2.22 mmol/l) threshold (11). Yet, failure to achieve a satisfactory glucose nadir (i.e. at, or below 60–62 mg/dl) during GST might indicate a suboptimal stimulus for GH secretion. For instance, such situation was observed in four subjects (4/27 i.e. 15%) from our patients with a history of pituitary disease, but an intact HPA axis, who failed obtain glucose concentration at, or below 62 mg/dl with GH concentrations between 1.07 and 3.0 ng/ml. Similar situation was observed in four Controls – 5.2% (i.e. GH between 3.0 ng/ml and 1.07 ng/ml, glucose nadir above 60 mg/dl), while further four Controls (5.2%) failed to reach 1.07 ng/ml GH cut-off with nadir of glucose concentrations above 60 mg/dl. Though it is difficult to extrapolate our data to larger populations, we might speculate that between 5–10% of subjects with an intact pituitary function (GH cut-off depending) might fail to demonstrate adequate GH secretion, possibly in relation to failure to obtain an adequate glucose nadir during GST. In such settings an alternative stimulatory test designed to test GH reserve (such as ITT) should at least be considered. Hence, results of our study are important for all clinicians involved both in diagnosis and in treatment of GH-deficient individuals. GH treatment is associated with significant cost to all health-care providers, so that it is essential that a reliable test is used to either confirm, or refute GH-deficiency.

We also note that despite slightly higher BMI in patients with the history of pituitary disease there was no difference in glucose x insulin product at all time-points of GST, thus denoting no major differences in insulin resistance between investigated groups. There was, however, slightly greater decline in glucose concentrations versus time zero in controls (18.55 ± 12.57 mg/dl for controls versus 13.54 ± 8.23 mg/dl for pituitary patients, p=0.007), consistent with lower mean minimal glucose concentration during GST (p=0.0013) in the control group (**Table 3**). The reason for that is not entirely clear, however our pituitary patient group consisted of individuals who were both GH sufficient and insufficient, while to the best of our knowledge there are no data of the relationship between overall glucose decline during later part of GST and degree of GH-insufficiency. Such issue might be, however, a subjects of an another study.

In our opinion, our results are of interest despite the fact both BMI and age seem to be the most important determinant of GH secretion during GST. It is well recognized that BMI negatively influences GH secretion (16, 17), while GH secretion also decreases physiologically with aging (18). Yet, once these non-modifiable factors are eliminated, then it appears that glucose nadir during GST is another important parameter related to GH secretion during GST.

To the best of our knowledge, this is the first study that provides calculated numerical data as to what glucose nadir would provide an optimal conditions in order to ensure a satisfactory stimulus for GH secretion during GST.

We note that 1.07 ng/ml GH cut-off (14) is very similar to 1.0 ng/ml cut-off suggested for obese individuals by Dichtel et al. (16) and Cuboni et al. (17), where application of the lower GH cut-off (1.0 ng/ml) would not influence our results. Hence, we describe a similar threshold of glucose (62 mg/dl (3.44 mmol/l) for combined group of healthy subjects and subjects with history of pituitary disease, but intact HPA axis, who did not take any hormonal treatment. In our study we have used the standard UK 180 minutes GST protocol. It might be argued that some GST protocols extend the test up to 240 minutes, however, Leong et al. (3) demonstrated that 240 minute sample did not usually provide any extra information. The issue of an optimal duration of GST as well as the number of time points is a subject of debate (16), where some authors (19) suggested even limitation of the test to 0, 150 and 180 minutes. In our previous study on copeptin secretion (20) we observed peak GH response at 150 minutes of GST. Some authors also comment that detailed analysis revealed that no subjects would be reclassified when GH concentrations from the last hour of the test (i.e. at 210 and 240 minutes) are excluded (17).

Another interesting (though negative) aspect of our study is a failure to find any parameter related to glucose or insulin fluctuations during GST and subsequent increase in cortisol concentrations during the second part of GST (initially during GST there is usually a small fall in cortisol concentrations, most likely related to diurnal variation, as all tests were performed in the morning). Activation of vasopressin (AVP) secretion by hypoglycaemia was postulated as one of the mechanisms involved in GH and cortisol secretion during ITT (21, 22). Somatostatin infusion was shown to decrease AVP secretion during ITT despite similar glucose nadir (23, 24), while AVP and oxytocin responses during ITT were completely abolished when the concomitant infusion of glucose prevented insulin-induced hypoglycemia (25). Simultaneously, the above authors demonstrated that hyperinsulinaemia is not involved in AVP release.

In this aspect we would like to briefly comment on the cortisol cut-off applied in our study (13.6 ug/dl=377 nmol/l). It is now universally accepted that the original cut-off point established by Plumpton and Besser in 1969 (for an ITT and later extrapolated into GST) (26) was too high, while modern cortisol assays yield readings by average about 50 nmol/l lower than in the 70-ties. Indeed 21 individuals from the control group (27.3%) in our study, failed to reach a 18 µg/dl (500 nmol/l) cortisol cut-off point. Hence, there is a tendency to use lower cut-off points than originally described. We used cortisol concentration cut-off of 13.6 ug/dl (377 nmol) as suggested by Yo et al. (12). This cut-off point effectively predicts a 100% pass on short synacthen test, and is close to the lower ITT cortisol cut-off of 416 nmol/l, recently suggested by Lazarus et al. (2). Interestingly, even a lower cortisol cut-off point of 11.2 ug/dl (310 nmo/l) during GST was suggested by Hamrahian et al. (27). We need to stress, however, that our study was not designed to investigate the optimal cut-off points both for cortisol and GH, but to investigate a relationship between these variables, i.e. the variations of glycaemia and/or insulinaemia and secretion of both GH and cortisol during GST.

As AVP and copeptin are secreted in equimolar amounts, but copeptin is much more stable and much more easy to measure (28), current studies usually utilize copeptin measurements instead of AVP. An increase in AVP/copeptin has been confirmed to occur during ITT (29, 30), while we have demonstrated that activation of AVP/copeptin secretion is also present during GST (20). Our observation was subsequently confirmed by others (31–33). In the setting of glucose fluctuation during GST with relative hypoglycaemia in the latter part of the test, we postulated a possible involvement of AVP in ACTH-cortisol and GH secretion during GST. Nevertheless, in another study (34) we demonstrated that intramuscular glucagon is capable to stimulate secretion of copeptin and GH, even without any concomitant increase in ACTH and cortisol. On the other hand, Atila et al. (32) demonstrated that a decrease in glucose levels during GST was associated with AVP/copeptin increase (r=0.53, p<0.01). All these observations point to a conclusion that AVP/copeptin might be potentially implicated in GH secretion, but the role of AVP in stimulation of ACTH/cortisol secretion appears to be limited. Hence, precise mechanism involved in stimulation of ACTH-cortisol secretion during GST still remains to be elucidated.

For completeness, it should be noted, however, that AVP is certainly not the only factor potentially responsible for GH secretion during GST, as release of an active peptidyl fragment from glucagon proteolysis after intramuscular injection was also postulated. For instance Arvat et al. (35) demonstrated that the combined administration of glucagon and hexarelin (a GH secretagogue) has a true synergistic effect on somatotroph secretion but a less than additive effect on corticotroph secretion. The authors suggested that these stimuli act via different mechanisms to stimulate somatotrophs while they could have a common action on the HPA axis. Though we did not formally test what factors apart from glucose and insulin fluctuations might be involved in stimulation of GH secretion during GST, it seems the fall in glucose during the latter part of GST is not the only mechanism responsible for GH secretion during GST. This notion is supported by the fact that stimulation of GH and cortisol secretion occurs only after intramuscular, and not intravenous glucagon administration (4), though fluctuations of glucose and insulin occur in both cases. Indeed, intravenous glucagon test has been used for assessment of C-peptide reserve (36).

The limitations of our study include the lack of the second stimulatory test (such as an ITT that is still considered to be the gold standard) that could be used to assess GH secretion in subjects who failed to obtain either 1.07 ng/ml, or 3.0 ng/ml GH cut-off. We also emphasize that in our study there were only four subjects aged below 18 (and only one below 14 years of age all from Control group), so our results apply effectively to adults and cannot be automatically extrapolated to children investigated for possible GH-deficiency. Caution should be also taken regarding to subjects with an extreme obesity as in our study there were only three individuals with BMI above 40 kg/m^2^ (two pituitary patients and one from the Control group).

In summary, our study demonstrated that apart from BMI and age, the nadir of glucose concentrations during GST is the only variable associated with a magnitude of GH secretion (ΔGH), while optimal conditions for GH secretion are achieved if glucose falls at least down to 60–62 mg/dl (3.33–3.44 mmol/l), or below this concentration. We postulate that glucose concentrations should be measured during GST in order to ensure that adequate stimulation for GH secretion had been obtained. On the other hand, mechanisms responsible for stimulation of ACTH-cortisol secretion during GST still remain to be elucidated.

## Data Availability

The raw data supporting the conclusions of this article will be made available by the authors, without undue reservation.
